# A spatial evaluation of socio demographics surrounding National Priorities List sites in Florida using a distance-based approach

**DOI:** 10.1186/1476-072X-8-33

**Published:** 2009-06-17

**Authors:** Greg Kearney, Gebre-Egziabher Kiros

**Affiliations:** 1 Florida Department of Health, Division of Environmental Health, 4052 Bald Cypress Way, Bin A#08, Tallahassee, Florida, 32399-1712, USA; 2 Institute of Public Health, 209b Frederick S. Humphries Science Research Center Florida A & M University, Tallahassee, Florida, 32307, USA

## Abstract

**Background:**

Over the last two decades, various spatial techniques have been demonstrated using geographical information systems (GIS) to adequately estimate and characterize inequities of minority populations living near environmentally hazardous facilities. However, these methods have produced mixed results. In this study, we use recently developed variations of the "distance based" approach to spatially evaluate and compare demographic and socioeconomic disparities surrounding the worst hazardous waste sites in Florida.

**Methods:**

We used data from the 2000 US Census Bureau and the Florida Department of Environmental Protection to identify selected socio and economic variables within one (1) mile of 71 National Priorities List (NPL) or Superfund sites in Florida. ArcMap (ESRI, v. 9.2) was used to map the centroid locations of each of the NPL sites as well as identify and estimate the number of host and non-host tracts. The unit of analysis in this study was at the census tract level. Logistic regression (SAS v9.1.3) was used to determine if race/ethnicity and socioeconomic indicators are significant predictors of the location of NPL sites.

**Results:**

There were significant differences in race/ethnicity composition and socio-economic factors between NPL host census tracts and non-host census tracts in Florida. The percentages of Blacks (OR = 5.7, p < 0.001), the percentage of Hispanic/Latino (OR = 5.84, p < 0.001), and percent employed in blue collar occupations (OR = 2.7, p < 0.01) were significant predictors of location of NPL facilities.

**Conclusion:**

The recently developed distance-based method supports previous studies and suggests that race and ethnicity play substantial roles in where hazardous facilities are located in Florida. Recommendations include using distance-based methods to evaluate socio and demographic characteristics surrounding other less known environmental hazardous facilities, such as landfills, or Toxic Release Inventory (TRI) sites.

## Background

Since the release of Bullard's [1] landmark book, *Dumping in Dixie: Race, Class, and Environmental Quality*, there has been a proliferation of research in the US to describe and adequately characterize the socio and economic demographics of minority populations living near or areas surrounding environmentally hazardous facilities. However, during this time much of the research methods to spatially evaluate and demonstrate the disproportionate inequities among classes of race, economics and social characteristics have produced inconsistent study findings.

A majority of these environmental justice studies have been based upon using census data to compare socio demographic and socioeconomic characteristics of people living within defined geographic host units with those living in non-host units. These traditional techniques typically implied that people living in a host unit were located more closely to a hazardous facility than those people living in a non-host unit. This is an inaccurate assumption and a weak method for spatially examining demographics; considering that the facility could be located at the boundary of the host unit, while the majority of the population lives adjacent to the site in the neighboring non-host unit. Mohai and Saha [2] describe that the spatial variation in the analysis of socio and economic demographics that exists in most geographical studies are primarily due to the different methods used to assess the hazard and the surrounding population.

In this study, we use recently developed distance-based methods [2,3] to evaluate and determine if race, ethnicity and socioeconomic indicators are significant predictors of NPL sites in Florida. The purpose of this research is to (1) apply recently developed distance based methods and spatial techniques for estimating socio demographic and economic characteristics around environmental hazardous waste sites in Florida; (2) review previous environmental justice studies and problems associated with using the unit hazard approach and, (3) examine the importance of race/ethnicity and socioeconomic factors on the location of NPL sites. We hypothesize that race/ethnicity and socio demographic variables are significant determinants of the location of NPL sites in Florida.

The Environmental Protection Agency (EPA) defines environmental justice as the fair treatment and meaningful involvement of all people regardless of race, color, national origin, or income with respect to the development, implementation, and enforcement of environmental laws, regulations, and policies [4]. Over the past two decades, an increased expressed interest among communities, government agencies, and researchers have provided well deserved attention to address environmental injustice and move this issue forward. However, in light of progress being made to recognize environmental injustices in communities with people of color, the variations of spatial methods to adequately evaluate socio demographic population characteristics near environmental hazards have produced mixed results. One of the overarching debates among researchers that have led to this disagreement stems from the use of different spatial methods for estimating populations and their socio economic and demographic characteristics. For example, studies by Anderton et al. [5] and Davidson and Anderton [6] found no statistical significance between minority populations and the location of environmental hazardous waste sites using a spatial analysis technique known as the "unit-hazard" coincidence method. By contrast, studies by Bullard et al. [3], Gragg et al. [7], Rinquist [8], identified racial and socioeconomic disparities to be associated with the location of hazardous waste sites when using alternative "distance based" spatial methods.

### GIS, Demographic Data and NPL's

The combination of geographic information systems (GIS) and demographic data have proven popular among researchers for evaluating population characteristics around environmentally hazardous facilities. In recent years, the features and spatial tools within GIS software packages have made considerable advancements for providing researchers with the ability to demonstrate where people live in relationship to hazards in their community. Common data sources that researchers turn to for demographic data are either from the US Census Bureau or purchased through private companies. Data from the US Census offers several advantages; it is free; available at different geographical levels (for example, block group, census tract), and easily downloaded from the Internet http://www.census.gov/. Census data can be imported and used in combination with GIS to assist researchers and public health professionals with evaluating socio economic and demographics of minority populations around environmental hazards such as the US EPA NPL sites.

The NPL sites are considered the worst hazardous waste sites in the US and pose the greatest human health risk. In December, 2008, there were approximately 1,300 NPL sites scheduled for clean up, with Florida ranking 6^th ^in the nation with 52 active sites. According to the Agency for Toxic Substances and Disease Registry (ATSDR), approximately 11 million people, including 3–4 million children, live within one-mile of an NPL site with about half of these facilities presenting a hazard to human health [9].

### Spatial methods for estimating environmental injustice

Bullard et al. [3] describes two primary types of spatial modeling techniques that are used when conducting environmental justice analyses of socio economic and demographics around environmental hazards; 1) the classical, "unit-hazard coincidence model," and 2) the newer, "distance-based" approach. The traditional unit-hazard coincidence model is the most widely used approach for assessing demographic disparities in the distribution of environmental hazards and is also the weakest in its ability to control for the proximity between such hazards and nearby populations [2].

**Figure 1:**
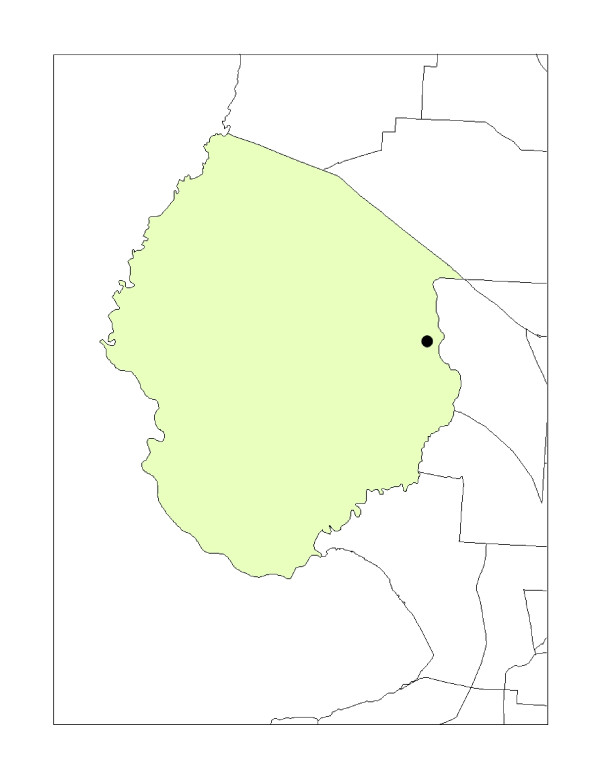
**Unit-hazard coincidence model: A NPL site located in a host census tract**.

The classical unit-hazard method is quite simple. First, a predefined geographical unit of analysis is selected. Examples of such geographical units include a county, zip code, block group or census tract where the hazardous waste site is located. Second, a comparison unit of analysis is chosen, typically, a unit without the hazardous waste site. Third, the demographic characteristics between the different geographical units are compared. The unit-hazard method allows for a straightforward approach for quickly calculating and comparing geographical areas, but offers several limitations. First, the method does not take into account the proximity of the hazard location with respect to the surrounding units. As shown in Figure 1, if a hazard is located near or at the edge of a census tract the adjoining unit's population demographics in the adjacent census tract(s) are not taken into account, therefore, could be considered misleading.

Second, the geographic location and the sheer size of the unit can distort the results by either over or under representing the demographic characteristics in the host tract where the hazard is located. Although limited, the traditional unit-hazard model has been the most typical and widely used method for many previous environmental justice studies. In 2006, a national environmental justice evaluation by Mohai and Saha [2] described and compared the unit-hazard coincidence model with "newer," distance based approach methods. As noted, the authors found the unit hazard approach was limited by the ability to control for the proximity between environmental hazardous sites and nearby residential populations. This limitation occurs primarily by the failure to use the exact location of where the environmental hazard is located within the host tract, and failure to control for proximity to neighboring geographic units [2]. Failing to identify the location of the facility within the host tract can result in inadequately capturing surrounding demographics in host and non-host tracts. The limiting assumption is that people living in host tracts are located closer to the environmental hazard than those living in non-host tracts.

**Figure 2:**
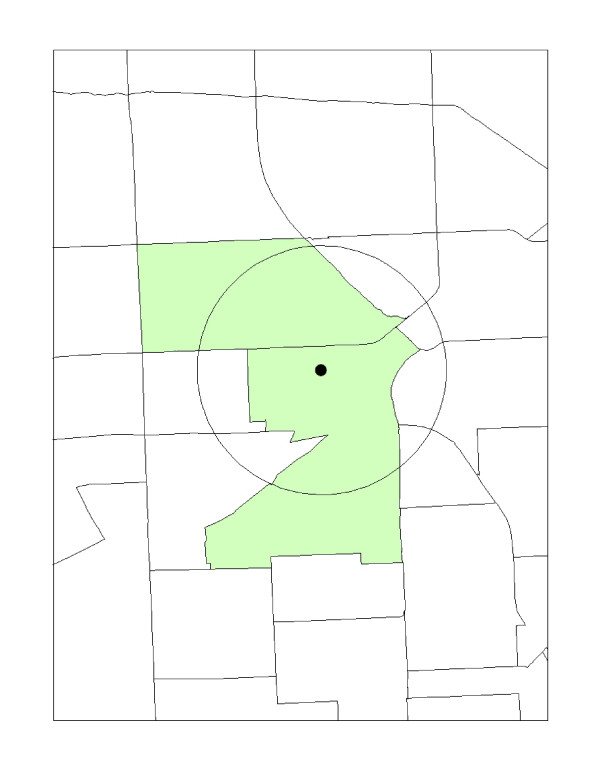
**Centroid containment method**.

In a recent study, Mohai and Saha [2] discuss and demonstrate the limitations of the unit-hazard model, and as an alternative offer the distance-based approach as a more accurate estimate for spatially examining demographics around hazardous facilities. Several spatial variations of the distance based method exist and includes the following: a) the distance boundary intersection method, b) the centroid-containment, (also known as the 50% areal apportionment method), and c) the areal apportionment method. As shown in Figure 2, the centroid containment, or 50% areal apportionment method sets a predefined circle or buffer (e.g. one-mile) around an environmental hazard, such as a hazardous waste site. If the circle captures the geographical center point, or at least 50% of the surrounding unit's geographical center(s), then it is included as part of the host neighborhood. All other geographical units outside of the host neighborhood, where 50% or less is captured, are excluded. In the event the environmental hazard's location lies outside the circle of its own host unit's geographic centroid, it is not included. The centroid containment method can be computed by the following formula [2]:

where, C_aggregated _is the aggregated socio or economic variable of the geographical unit; *(p) *is the population of the unit and *(c) *is the socio or demographic variable of the geographical unit.

**Figure 3:**
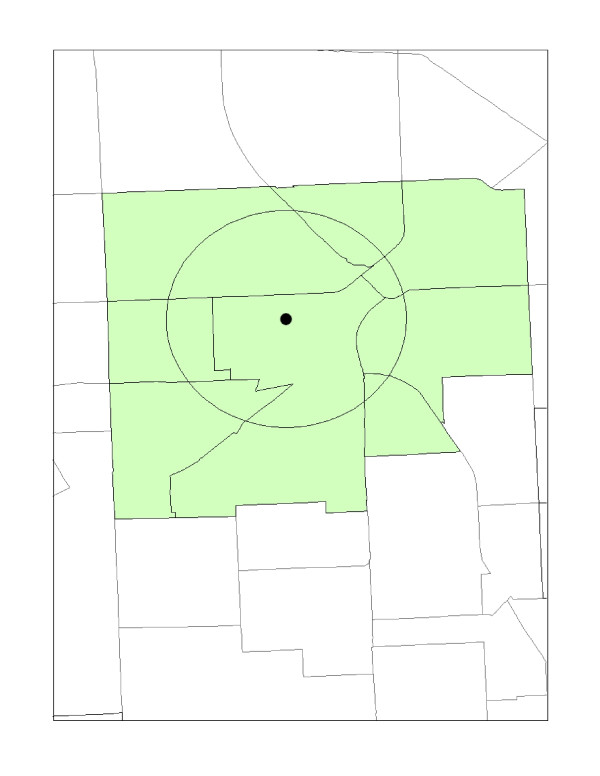
**Distance boundary method**.

As shown in Figure 3, a variation of the centroid containment method is the distance boundary method. Instead of capturing 50% of an area's unit, the distance based boundary method selects any units boundary that is either wholly contained, partially intersected or tangent to a specified distance, centered on the environmental hazard considered to be in the host neighborhood [2]. To further explain, this method includes the host tract and "contiguous" geographical areas that either share, or have a portion of its unit within a specified distance, as in this case, a one-mile distance from the host tract. This method differs from the unit-hazards approach because it only includes contiguous tracts within a specified distance. However, it shares the same proximity limitation as the unit hazard method because its geographic boundaries, such as census tracts, vary in shape and size. Therefore, this approach lacks the ability to control for demographic characteristics that may extend beyond the distance unit being considered. Because it lacks a control measure for proximity, the distance boundary method has been described by Mohai and Saha [2] as the least effective of the distance based approaches.

**Figure 4:**
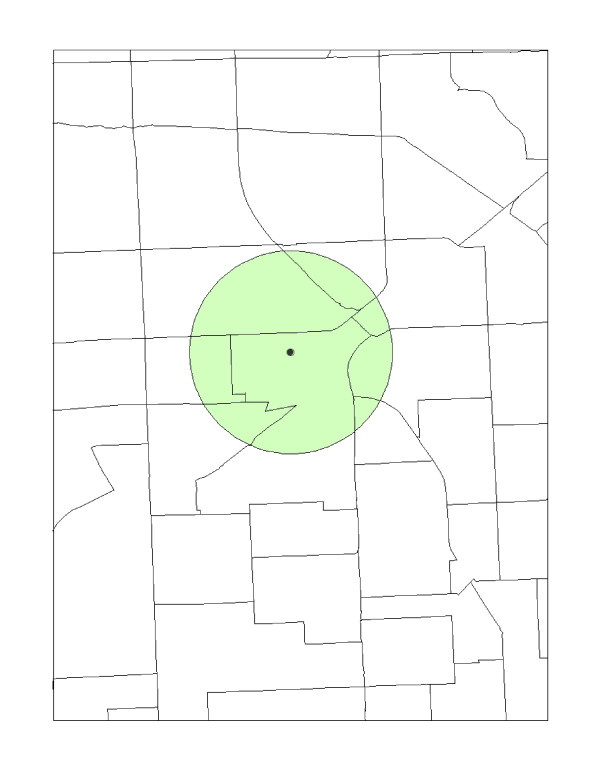
**Areal apportionment method**.

A third distance-based approach is the areal apportionment method. In this method, (shown in Figure 4), each of the geographical units characteristics are weighted based upon the proportion of the geographical unit that is captured by the area of the circle (or buffer) surrounding the environmental hazard. For example, if 35% of an adjacent geographic unit is included in the buffer, then that percentage is weighted by the population size. The units included in the buffer are aggregated and compared against the demographic units not captured. The averaged demographic characteristic can be computed by [2]:

where *(C) *is the demographic characteristic within the neighborhood's one-mile radius, *A *is the total area of the unit, *(a) *is a subset of the total area, *n *is the number of units, *(p) *is the population and *(c) *is the socio or economic variable of the unit.

The aims of this project were multi-fold. First, we wanted to characterize the socio and economic characteristics around NPL sites in Florida using each of the distance based methods previously described. Next, we wanted to select one of the distance based methods to determine if race/ethnicity and selected socio variables were predictors of the location on NPL sites. To accomplish this, we identified and selected census variables that were most commonly used in previous environmental justice studies. Those variables included, race, ethnicity, families living in poverty, plumbing facilities, renter occupied, foreign born, occupation and education (See Table 1).

**Table 1:** Census variables used to evaluate demographic and economic characteristics around NPL sites

Name	Attribute
Race	Persons who are Black, American Indian, Alaskan Native, other Pacific Islander, Asian, Native Hawaiian, some other or two or more races

Families Living in Poverty	Families income in 1999 below poverty

Hispanic or Latino Origin	Persons who identify themselves as, Mexican, Puerto Rican, or Cuban as well as those who indicated that they were "other Spanish, Hispanic, or Latino."

Median Income	Income below $41,994 in 1999

Lacking Complete Plumbing Facilities	Lacking all of the following; hot & cold piped water, flush toilet and a bathtub or shower (in the housing unit)

Renter Occupied	Occupied units which are not owner occupied, whether rented for cash rent or without payment of cash rent

Occupation (Includes white collar and blue collar trades)	Persons 16 and over employed in management, financial, business and related professional occupations; Persons 16 or older employed in farming, agriculture, forestry, mining, construction, manufacturing, wholesale, retail trade, transportation, utilities, food service or other laborer type occupations

Total Population	All people, male, female, child, and adult living in a given geographic area

Education	Persons 18 or older with a high school diploma or less education; bachelor degree; and graduate or professional degree

Foreign Born	Persons who were born outside of the US, includes, naturalized and non-citizens.

We selected a one-mile radius from the center of each of the 71 NPL sites and compared the variables above in NPL host tracts to those in non-host tracts using the three distance-based approaches. We then used a multivariate logistic regression to examine if race/ethnicity and socioeconomic indicators have any significant effect to predict the location of NPL sites. This is the first known published study to use distance based methods with the 2000 US Census Bureau data at the census tract level to evaluate race and ethnicity while controlling for socio demographics and economic variables surrounding NPL sites in Florida. Previous studies by Gragg et al. [7] and Strensky and Hogan [10], have evaluated socio demographics surrounding NPLs and other environmentally hazardous facilities in Florida. However, as far as we know, this is the first known study focusing specifically upon demographics surrounding NPL sites in Florida using the data from the 2000 US Census Bureau.

## Methods

We selected 71 NPL hazardous waste sites that were considered *"active" *by the US EPA in Florida between 1985 and 2000. Active sites are defined as "whether a site assessment, removal, remedial, enforcement, cost recovery, or oversight activities are being planned or conducted under the Superfund program" [4].

We accurately identified and mapped the location of each site and captured the surrounding census tracts that were within one-mile of each of the 71 sites as previously described using the distance-based methods. We obtained two separate governmental data sets of longitude and latitude coordinates of the NPL site locations; (1) US EPA and (2) Florida Department of Environmental Protection (FDEP). We mapped the geographic coordinates of each NPL site provided by each agency, using ArcMap (ESRI, v. 9.2). Next, we cross verified and compared the locations of each NPL site using the longitude and latitude feature in Google Maps. In several instances we found differing geographical point locations between the two data sets for NPL sites. Upon further exploring with Google Maps, we found several of the US EPA NPL site coordinates were geocoded and mapped to the physical address, or location entrance of the facility; whereas, the FDEP coordinates were mapped to the geographic center of each NPL site location. We contacted FDEP, and staff confirmed that the longitude and latitude coordinates of each NPL site contained in their database were point-in-polygon centroids, (mapped to the center of each NPL site). More specifically, the NPL coordinates were established by using a combination of geo-referenced, digital aerial photos (with aerial accuracy of +/- 3 pixels (or +/- 7 ft., or whichever was greater), and property appraiser records.

Because the physical shape and land area of NPL properties vary considerably in size, we used the FDEP's geo-referencing points-in-polygons instead of polygons. The geo-referenced points of each site represented the center of each of the NPL property locations, whereby providing surrounding census tracts an equal chance of being captured in the defined one mile buffer rather than the center of each polygon. For example, Superfund sites in Florida vary from one to over 1000 acres in size. If the site was located near the edge of a census tract, the point-in-polygon method would accurately identify the geographical center of the environmental hazard location and capture surrounding demographics of neighboring tracts using each of the described distance based methods.

Demographic data on population, race, families living in poverty, Hispanic, median income, plumbing facilities, education, renter occupied housing, and occupation were downloaded from the U.S. Census Bureau, American Factfinder (Summary File 3 (SF3) website at http://factfinder.census.gov. We used ArcMap (ESRI, v. 9.2) to map the centroids location of each of the 71 NPL sites, and then identified all host census tracts within one-mile of each of the sites and non-host census tracts outside one-mile of the 71 NPL sites using each of the three distance based methods. For the areal apportionment method we used the "Clip" feature found in Arc Toolbox. For the boundary intersection and centroid-containment methods we used the "Select by Location" feature in ArcMap.

### Logistic Regression Analysis

We used logistic regression analysis (using SAS, v. 9.1.3) to determine if race/ethnicity and socioeconomic indicator variables were significantly associated with the location of NPL sites. The unit of analysis was individual census tracts (n = 3,154). The dependent variable was whether or not a census-tract hosted an NPL within one-mile radius and assumed a value of 1 if the tract hosted an NPL site within one-mile radius and a value of 0 if it did not. The independent variables included in the final model were race/ethnicity measured as percentage of Hispanic or Latino, percentage of African Americans, and percentage of Asian/Pacific Islander and socioeconomic indicators measured as percentage with four-year college education and percentage employed in professional "blue collar" occupations. All of these variables were measured at the census-tract level. Most of the socio-economic variables including, percentage of families living in poverty were highly correlated with race/ethnicity and hence were dropped from the final logistic regression model to avoid the problem of multi collinearity. For example, the correlation between families living in poverty and percent Black in a census tract was 0.69.

We performed the logistic regression analysis in two stages. First, we ran a bivariate logistic regression by fitting only one independent variable at a time. Then we fitted a multivariate logistic regression model that included the five independent variables listed above. We presented Wald's Chi-Square test to assess our models goodness of fit. In addition, McFadden's pseudo-R^2 ^was used to show how useful the predictor variables were as a group in predicting the dependent variable [11]. Pseudo – R^2 ^in logistic regression is analogous to the coefficient of determination (R^2^) used in linear regression although both do not have the same variance interpretation. We reported the odds ratios and their corresponding p-values. Type III Sums of Squares were used to interpret the results of the logistic regression.

For the logistic regression analysis we chose the areal apportionment method (one-mile radius) because it gave "weight" to every geographical unit inside the one-mile distance buffer in constructing the host tract. Unlike the centroid containment method that uses the center of the unit as the determining factor to include in the host tract, the areal apportionment method provided weight to the proportion of geography that was captured in the buffered area. This provided assurance that the surrounding demographics, no matter how small of an area in the unit, were included in the host tract. A recent study by Bullard et al. [3] demonstrated that the centroid containment and areal apportionment methods lead to similar estimates when investigating racial and socioeconomic disparities within specified distances surrounding hazardous waste facilities.

## Results

**Figure 5:**
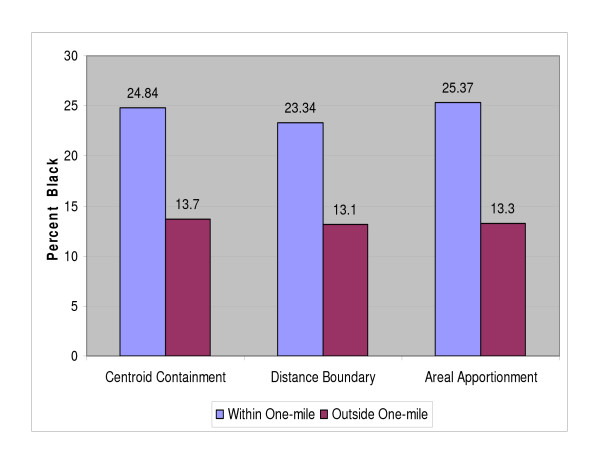
**Percent of Blacks within one-mile and outside one-mile of NPL sites**.

**Figure 6:**
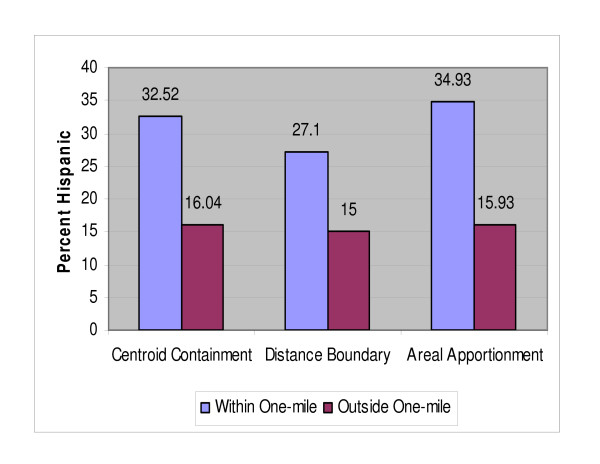
**Percent of Hispanics within one-mile and outside one-mile of NPL sites**.

As shown in Table 2, the socio economic and demographics from the US Census were mapped and compared using the three distance-based methods. Each of the three distance-based methods produced varied results. A higher percentage of Blacks, Other, and two or more races were found in NPL host tracts, than those found within non-host tracts. As shown in Figure 5 the percentage of Blacks were approximately 12% higher and Hispanics 19% (Figure 6) higher within host tracts compared to those of non-host tracts. In all three methods, the percentages of renter occupied housing, foreign born, and houses lacking plumbing facilities were higher in host compared to non-host tracts. The percentages of those in blue collar jobs were higher in the host tracts in the distance boundary and areal apportionment method, and the percentage of those holding a high school diploma was 3.64% higher in host tracts in the areal apportionment method, but lower for those holding a bachelor's degree, graduate or professional degree. It should be noted here that these results are estimates produced using the different methods and like any other estimate are prone to errors. Hence, it is difficult to determine with a certain degree of confidence which of the three methods produced better estimates.

**Table 2:** Demographic comparisons of census tracts within one-mile distance surrounding 71 NPL sites in Florida

	Centroid Containment Method		Distance Boundary Method		Areal Apportionment Method	
	Host Tracts (77)	Non Host Tracts (3077)	Host Tracts (62)	Non Host Tracts (3092)	Host Tracts (253)	Non Host Tracts (2901)
**Population**						
						
% Population	8.08	91.91	9.23	90.78	0.31	97.43
						
**Race/Ethnicity**						
						
% Black	24.84	13.70	23.34	13.10	25.37	13.30
% Native Am	0.34	0.34	0.34	0.34	0.31	0.33
% Asian	1.51	1.64	1.44	1.66	0.18	1.61
% Hawaiian	0.06	0.05	0.06	0.05	0.05	0.05
% Other Race	3.83	2.87	3.57	2.83	3.75	2.80
% Two or more Races	3.20	2.30	2.90	2.26	3.21	2.23
Hispanic	32.52	16.04	27.10	15.00	34.93	15.93
						
**Socioeconomic** **Characteristics**						
						
% Families Living in Poverty	15.09	8.76	13.00	8.50	4.65	8.73
Below Median Income	84.02	62.03	74.00	62.00	74.00	62.00
% Renter Occupied Hsng	43.80	29.42	36.96	29.17	36.10	29.38
Foreign Born	30.42	16.19	25.67	15.75	21.78	16.11
Lacking Plumbing	0.93	0.59	0.80	0.58	1.00	0.58
						
**Occupation**						
						
% White Collar	56.33	45.98	48.17	56.75	44.57	56.40
% Blue Collar	43.67	54.02	51.83	43.25	55.29	43.60
						
**Educational Attainment**						
% With High School Diploma	27.73	28.56	28.24	28.66	27.51	23.87
% With Bachelors Degree	18.52	19.02	17.72	19.11	16.92	18.58
% With Graduate or Professional Degrees	5.62	7.58	5.83	7.67%	5.36	7.93

Notes: Based on 2000 US Census Data STF 3. Education: Total Males and Females 18 years or older. 1999 Median Income in Florida was $41,994 dollars (US Census, 2000). Centroid containment method contained only those census tracts that had a geographic center within one-mile of an NPL. If the one-mile radius did not include the centroid of the host tract, it was not included. Occupation; % of White collar includes P50, % of Blue collar includes p50 (includes food service). Percentages differ because they do not include service protection services which could be considered white or blue collar.

Table 3 presents both unadjusted and adjusted odds ratios predicting the effects of race/ethnicity and socioeconomic indicators on the location of NPL sites in Florida. The overall model fit was good (*X*^2 ^= 123.38, df = 5, p < .001) and the pseudo-R^2 ^value (0.162) confirmed that the model was satisfactory in predicting location of NPL sites in Florida. The percentages of Hispanic/Latino, Blacks, and those employed in blue collar occupations were significant predictors of location of NPL facilities in Florida. More specifically, even after controlling for college education and working in blue collar occupations, the odds of a facility being located within a one-mile radius increased as the percentage of Hispanics/Latinos increased. The percentage of Asian/Pacific Islander decreased the likelihood, but not significantly. In addition, blue collar occupation remained a significant predictor in the multivariate logistic model. Interestingly, the percentage with four year college education (or Bachelor's degree) was not significantly associated with location of NPL sites in Florida in both the bivariate and multivariate models. It should also be noted that the unadjusted odds ratio shows that percent of people living in poverty is significantly associated with higher odds of living near host sites, but was dropped from the multivariate model because of the problem of multicollinearity.

**Table 3:** Comparison of independent effect of race/ethnicity on NPL location using areal apportionment method

Characteristics	Odds Ratio	95% CI	P-Value
Race/Ethnicity			
% Hispanic or Latino	5.853	3.446, 9.943	< 0.001
% African-American	5.746	3.422, 9.648	< 0.001
% Asian/Pacific Islander	0.314	0.056, 1.770	0.189
			
SES Indicators			
% with 4-Year College Education (Bachelor's Degree)	0.980	0.894, 1.074	0.662
% Employed in Professional "Blue Collar" Occupations	2.698	1.254, 5.806	0.011
			
-2 Log Likelihood	1910.053		
Model X^2 ^Test (df = 5)	123.38		< 0.001

## Discussion

A key finding from Bullard et al., *Toxic Waste and Race at Twenty: 1987–2007 *report [3], is that the newer, distance-based methods better determine where people live in relation to where hazardous waste sites are located. At the national level, African Americans, Hispanics/Latinos and Asian Americans/Pacific Islanders alike are disproportionately burdened by hazardous wastes in the U.S. and people of color and persons of low socioeconomic status are still disproportionately impacted and are particularly concentrated in neighborhoods and communities with the greatest number of facilities [2]. A study by Mohai and Saha [2] illustrates this point by using distance-based methods by demonstrating that race continues to be an independent predictor of where hazardous wastes are located, and a stronger predictor than income, education and other socioeconomic indicators. Using the described distance-based methods, our spatial evaluation demonstrated that race and ethnicity were significant predictors of NPL sites in Florida. However, the collinearity between race and socioeconomic indicators are a limitation of our study, as we were unable to include important measures such as poverty and median income into our final model because these variables were highly correlated with proportion Black. Therefore, we have to be cautious when comparing our findings with other previous studies that have considered more socioeconomic indicators.

One of the underlying limitations of a spatial study of this nature is that research findings vary with the geography chosen, and have been properly identified as the modifiable aeral unit problem (MAUP) [12]. The MAUP arises from scale, aggregation and the geographical data by a defined geographical boundary, such as a census enumeration district, to present results in more of a spatial phenomena [13]. The MAUP could be considered an argument for differing results. Stretsky and Hogan [10] pointed out that the geographical units of analysis may be problematic in the study of environmental justice, basically because of a tendency toward aggregation bias. We recognized this as an issue, and in our research, found the census tract to be the most commonly agreed upon geographical unit of spatial analysis for environmental justice studies.

Another questionable spatial concern rests upon our choice of using the points-in-polygon method rather than the polygon approach. We found that the points-in-polygon method worked well in most cases when buffering around NPL's, particularly when the site was located in a small to medium sized census tracts. However, when an NPL was located in a large census tract where the one mile buffer radius did not extend past the census tract boundary, it did not take into account any neighboring demographics, even if the extent of the boundary was located slightly inside the host tract buffer. As an alternative to this method, a comparative analysis of the surrounding demographics at different buffer radius's (e.g. one-mile, three-miles and five-miles) could be used to address this issue. In addition, the areal apportionment method worked well for capturing neighboring tracts within the defined buffer radius; however, it shares a similar limiting assumption as other methods including the unit hazard approach. It assumes an equal distribution of population characteristics across the geography, such as in this case, the census tract. As an alternative, future studies could address this issue by using the population-weighted based approach. Typically, this method considers the size and location of the population (e.g. towns, cities, tribes, etc.) rather than assuming a uniform population distribution across the geography. Also, using the distance based approach to evaluate racial and ethnicity around other, lesser known environmental hazards, such as landfills, or toxic release inventory (TRI) facilities could be evaluated in future studies.

## Conclusion

As GIS mapping techniques and research methods continue to improve, the choice of an "ideal" spatial method may surface among researchers. Nonetheless, the combination of new methods and advancements in GIS technology are highly important steps for researchers and public health officials to use to evaluate the socio demographics of communities living near environmentally hazardous facilities. It is anticipated that this research will have meaningful contribution to the existing literature and provide as a spring board for future in-depth studies to more closely examine racial disparities surrounding environmentally hazardous facilities in Florida and elsewhere.

## Abbreviations

ATSDR: Agency for Toxic Substances and Disease Registry; CT: Census Tract; FDEP: Florida Department of Environmental of Protection; FDOH: Florida Department of Health; NPL: National Priorities List; US EPA: United States Environmental Protection Agency.

## Competing interests

The authors declare that they have no competing interests. The views and opinions expressed in this manuscript are solely those of the authors and do not represent of that of the Florida Department of Health or Florida A & M University.

## Authors' contributions

GK and GEK conceived the study, participated in its coordination and execution, and wrote the manuscript. GEK created the variables and performed the statistical testing.

GK formatted the data and performed all the mapping. All authors read and approved the final manuscript.

## References

[B1] Bullard RD Dumping in Dixie: Race, Class, and Environmental Quality 1990 Boulder, CO: Westview Press

[B2] Mohai P Saha R Reassessing racial and socioeconomic disparities in environmental justice research Demography 2006 43 2 383 399 10.1353/dem.2006.0017 16889134

[B3] Toxic Wastes and Race at Twenty: 1987–2007: Grass roots struggles to dismantle environmental racism in the United States http://www.ejrc.cau.edu/TWART-light.pdf

[B4] U.S. Environmental Protection Agency http://www.epa.gov/oecaerth/environmentaljustice/index.html 11645578

[B5] Anderton D Anderson A Oakes J Fraser M Environmental Equity: The Demographics of Dumping Demography 1994 31 229 48 10.2307/2061884 7926187

[B6] Davidson P Anderton D The Demographics of Dumping II: Survey of the Distribution of Hazardous Materials Handlers Demography 2000 37 461 66 10.1353/dem.2000.0002 11086571

[B7] Gragg RD Christaldi RA Leong S Cooper M The Location of community demographics of targeted environmental hazardous sites in Florida Journal of Land Use and Environmental Law 1996 12 1 1 46

[B8] Ringquist E Assessing Evidence of Environmental Inequities: A Meta-Analysis Journal of Policy Analysis and Management 2005 24 2 223 247 10.1002/pam.20088

[B9] Agency for Toxic Substance and Disease Registry: Children's Health http://www.atsdr.cdc.gov/child/

[B10] Stretesky P Hogan M Environmental Justice: An Analysis of Superfund Sites in Florida Social Problems 1998 45 2 268 287 10.1525/sp.1998.45.2.03x0169m

[B11] Hardin JW Hilbe J Generalized Linear Models and Extensions 2007 Second College Station, TX: Stata Press

[B12] Liu F Environmental Justice Analysis: theories, methods and practice 2001 Boca Raton, FL: CRC Press

[B13] Unwin DJ GIS, spatial analysis and spatial statistics Progress in Human Geography 1996 20 540 551 10.1177/030913259602000408

